# Exome Sequencing of a Type 1 Diabetes Mellitus Family Exposes Both Common and Individualized Rare Variants Contributing to Pathogenesis

**DOI:** 10.1155/jdr/3346256

**Published:** 2025-12-02

**Authors:** Tomader A. M. Ibrahim, Rayan S. Ali, Mohamed A. Abdullah, Muntaser E. Ibrahim

**Affiliations:** ^1^Department of Molecular Biology, Institute of Endemic Diseases, Medical Campus, University of Khartoum, Khartoum, Sudan; ^2^Department of Paediatrics, Faculty of Medicine, Medical Campus, University of Khartoum, Khartoum, Sudan

**Keywords:** common variants, Diabetes Type 1, exome sequencing, individualized, MicroRNA, rare variants

## Abstract

Type 1 diabetes mellitus (T1D) is a disease of complex inheritance where genetic, immunological, and environmental factors interact in rendering the ultimate phenotype. To gain insights into the molecular etiology of the disease in a subset of a population that is sparsely investigated in genetic terms, like in Africa, exome sequence data from a T1D multicase family and a T1D cohort were investigated. The exome analysis identified several candidate genes related to T1D, like human leukocyte antigen (HLA), insulin (INS) gene, Cytotoxic T-lymphocyte–Associated Protein 4 (CTLA4), Protein Tyrosine Phosphatase Nonreceptor Type 22 (PTPN22), and Interferon-Induced Helicase C Domain 1 (IFIH1). A total of eight pathways were significantly overrepresented (*p* value ≤ 0.05) in target lists analyzed, including WNT, MARS2, TARS, STK36, TYR, TP73, ATIC, and HNF4. Based on Condel functionality scores and centrality positions in genetic interaction networks, two prominent candidates in diabetes mellitus and maturity-onset diabetes of the young (MODY)—HNF1A rs2464195 and HNF4A rs147638455—were identified. The two candidate variants were subsequently genotyped for further replication in a total of 47 T1D cases and 20 unrelated controls. No significant differences were observed (*p* = 0.73 and *p* = 1), as the variants turned out to be relatively common among Sudanese and absent or rare in a global sample. Expression analysis of these loci was carried out alongside two miRNAs, miR-105 and miR-518, which were selected based on in silico prediction (*p* = 0.057 and 0.038, respectively). The results revealed profound miRNA differential expression between T1D cases and controls, suggesting a role for miRNA in the regulation of susceptibility networks, but also the existence of within-family differences in the fold change. Such differences, especially if taken in connection with the clinical differences encountered in this family and the population variation, highlight the potential of both population-based and individualized approaches in fathoming underlying causes of pathogenesis leading to a T1D phenotype.

## 1. Introduction

Type 1 diabetes mellitus (T1D) is a multifactorial disease of complex inheritance that results from the interplay between environmental and genetic factors that does not fit any simple pattern of inheritance [1]. Familial aggregation and twin studies supported the aforementioned [2], and families with sibling risk are approximately 10 times greater to have T1D than in the general population [3]. This contrasts with Type 2 diabetes, where the sibling risk ratio is relatively modest at 3.5 [4]. Several significant genetic association studies shed light on the genetic complexity and multifactorial nature of T1D. These studies aimed at identifying genetic loci or regions associated with an increased risk of developing T1D. For instance, Onengut-Gumuscu et al. conducted one of the largest genetic association studies on T1D in 2015 [5]. The researchers were able to identify 44 genetic loci associated with T1D risk. More recently, Robertson et al. further expanded the list by conducting a large-scale genetic association analysis with approximately 60,000 participants [6]. The researchers identified 78 significant associations, including 36 novel ones. Unlike monogenic inheritance, in diseases of complex and polygenic inheritance like T1D, identifying the true culprit within the occurring genetic change might prove cumbersome [7]. Comprehending the potential spectrum of variants that may have contributed to pathogenesis may prove challenging, particularly in populations with complex allele structure and different homozygous reference variants [8]; establishing whether these variants are rare or common is believed to be key. Disease-causing variants are generally of low allele frequency, with the exception of cases where heterozygous advantages result in increased frequency of pathogenic variants. Another challenge is condemning these variants to function as some recent reviews differentiate between damaging and pathogenic variations [9]. Applications of recent molecular genetics strategies, like next-generation sequencing, promise a circumvention of some of these inherent hurdles, fostering a better understanding of susceptibility to diabetes mellitus. This includes identifying basic etiology and possible complications of which will be key in predicting future development of associated clinical features and allowing for early preventative or supportive treatment [7]. For example, oral sulfonylurea treatment of HNF1A/HNF4A-MODY requires genetic testing to diagnose the condition [10]. Also nowadays, personalized approaches in medicine that place individual variation through genomic and other biomarkers in a position to predict risk of disease or response to therapy are being advocated [11]. With such a background and based on the above, the current combined investigation of a multicase family and a T1D cohort from Sudan attempts to unravel some of the unique features in the molecular etiology of the disease in a population that has been sparsely investigated for T1D and to better our understanding of its basic biology.

## 2. Subjects and Methods

### 2.1. Ethics Statement

This research was approved by the ethics committee of the Institute of Endemic Diseases, University of Khartoum. All subjects signed an informed written consent.

### 2.2. Study Groups

**Figure 1:**
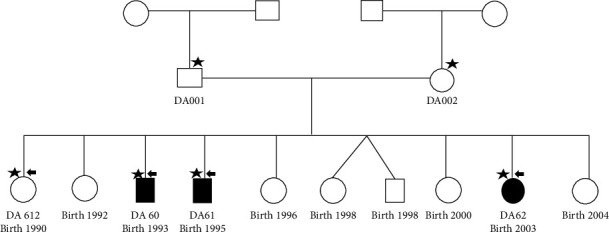
Family pedigree of the current study with the three T1D children investigated (shaded symbols denote affected probands). Those chosen for exome sequencing are indicated by black arrows. Black stars indicate family members who donated blood for genotyping and quantitative real-time PCR.

The study sample consisted of a cohort of 47 individuals suffering from T1D recruited from the Sudan Childhood Diabetes Center in Khartoum and 20 healthy individuals without family history as controls. In addition, one typical T1D Sudanese family with three siblings diagnosed with T1D and three healthy controls, “parents and an unaffected daughter,” were enrolled (Figure 1). Two of the cases (DA60 and DA62) were complaining of celiac disease, in addition to thyroid problems, while the third suffered from T1D only.

In this study, all diabetic patients use insulin (INS) injections for diabetes management, which aligns with standard practice for T1D treatment in Sudan.

Serum was obtained from the blood of the target family members (three cases and one control) and was sent for clinical chemistry tests (C-peptide, GAD, thyroid function, and celiac test).

### 2.3. DNA Extraction and Measurement

DNA was extracted using a guanidine-based method. The DNA purity was measured by NanoDrop (Spectrophotometer ND-1000), for the absorbance at the 260/280 nm ratio. DNA concentration was ≥ 200 ng/*μ*L, and purity (260/280 nm ratio) ranged between 1.8 and 2.2.

### 2.4. Whole-Exome Sequencing and Data Analysis

Exome sequencing of the multicase family included three T1D cases and one unaffected control (indicated in Figure 1) and was carried out using Illumina HiSeq 2000 (Illumina, BGI, Hong Kong). Bioinformatics analysis was performed for the exome data using different bioinformatics tools. Mutations were classified and filtered as deleterious or neutral using the CONsensus DELeteriousness (Condel) score of missense SNVs (https://omictools.com/consensusdeleteriousness-score-of-missense-snvs-tool). SIFT scores less than 0.05 and/or PolyPhen-2 scores above 0.85 were used to predict whether an amino acid substitution affects the function or structure of a protein. Classification also included whether it is a conservative change and whether it is predicted to be benign by multiple in silico algorithms and/or has population frequency not consistent with disease (https://clinvarminer.genetics.utah.edu/submissions-by-2variant/NM_000545.6%2528HNF1A%2529%253Ac.1501%252B7G%253EA). To determine the relationship and overlap between diseases caused by 46 damaged genes extracted from exome data, various online tools like Enrichr (http://amp.pharm.mssm.edu/Enrichr/#find) and ConsensusPathDB-human (http://consensuspathdb.org) were used. A network of microRNAs (miRNAs) was also constructed based on significant differences, selected by the TargetScan miRNA database within Enrichr. In addition to constructing gene networks, the Kyoto Encyclopedia of Genes and Genomes (KEGG) pathway database was utilized to understand high-level functions and utilities of the biological system (http://www.genome.jp/kegg/pathway.html). Untranslated Region 3 (UTR3) of exome data was analyzed using PolymiRTS Database v 3.0 (http://compbio.uthsc.edu/miRSNP/) to detect the degree of damage among the variants and the associated miRNA of each variant to T1D. DIANA software was used to assess the target genes; the prioritized miRNAs, miR-105, and miR-518; and the pathways for each gene. Variants were filtered out in the dbSNP135 and 1000 Genome databases (https://www.1000.genomes.org). SNP's selection for subsequent analysis was based on the preliminary results obtained from the exome data.

### 2.5. Polymerase Chain Reaction (PCR) and Restriction Fragment Length Polymorphism (RFLP)

Both regions, which contain target variants, were amplified using PCR. All PCR products were subject to digestion using SPMF1 and MNX1 enzymes. The three affected siblings with T1D and three family controls were analyzed in addition to 47 cases from the Jaber–Abulez diabetes center in Khartoum with T1D and 20 healthy controls from our DNA bank, as verification for the involvement of HNF4A and HNF1A in the pathogenesis. Chi-square values of SNP's genotype distribution were also performed with Fisher's exact test, with significance values at *p* ≤ 0.05.

### 2.6. RNA Extraction, Measurement, Reverse Transcription, and Real-Time PCR (qPCR)

RNA samples were obtained from blood using the easy-BLUE kit according to the manufacturer's instructions (iNtRON Biotechnology, Inc.). Complementary DNA (cDNA) was transcribed with Maxime RT Premix Kit (iNtRON Biotechnology, Inc.). Quantitative PCR assays were carried out using 20 *μ*L reactions containing Power SYBR Green Master Mix (iNtRON Biotechnology, Inc.) with specific primers for each gene, in an ESCO Spectrum 48 real-time thermal cyclers. The Livak method of ΔCT was used to calculate the expression levels of HNF1A, HNF4A, miR-105, and miR-518.

## 3. Results

All three T1D siblings and the control, DA60, DA61, DA62, and DA612 (Figure 1), that were subject to exome sequencing, were also subject to the following investigations, with values indicated: random C‐peptide < 0.1 ng/mL and HbA1C percentage of 11.4%, 10.6%, 11.6%, and 6.5%, respectively.

All were negative for diabetes autoantibodies (GADA), and no family history of diabetes is known to them in at least three generations. Diagnosis for celiac disease was confirmed in two siblings (DA60 and DA62) based on specific immunological tests (Table 1). These differences in clinical phenotype between the T1D individual samples are also seen in the expression profiles in both miRNA and the candidate genes, as presented below. All initial and clinical investigations were followed according to T1D guidelines, have been under INS treatment from the time of onset, and have not shown a lack of response to treatment during this period.

**Figure 2:**
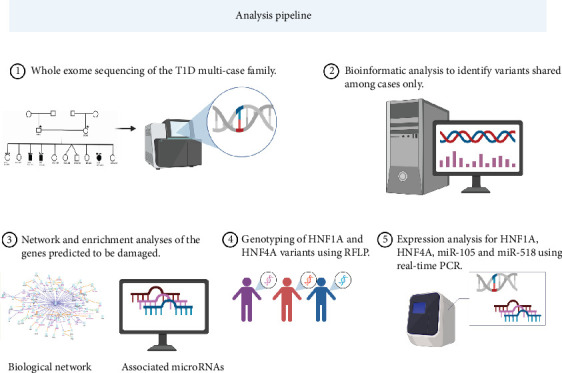
Graphical summary of study steps and different aspects of the workflow. Beginning with sample collections, exome sequencing, and application of different bioinformatics tools, and further investigations conducted for variants, HNF1A rs2464195 and HNF4A rs147638455 (genotyping and expression), in addition to microRNA (miR-105 and miR-518) expression.

The statistics of the identified variants in exome data are summarized in Figure S1, supporting information data, which describes the genetic variations and mutational types within the study samples, and a graphical summary of the study steps is demonstrated in Figure 2.

**Figure 3:**
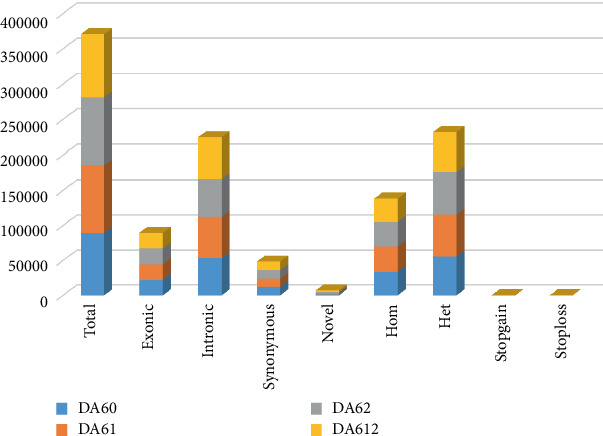
SNP analysis of the exome data showing the distribution length of reads for each sample. Exonic (coding exonic portion), Intronic (noncoding portion), Synonymous (change in the DNA sequence, but no change in the encoded amino acid), Novel (newly discovered), Homo (homozygous), Het (heterozygous), Stopgain (nonsynonymous SNV that lead to the immediate creation of stop codon), and Stoploss (elimination of stop codon at the variant site).

A total of 9000 variants were found to be shared between the three cases (DA60, DA61, and DA62), of which 175 were nonsynonymous. According to the SIFT score of less than 0.05, 48 SNPs were found damaging, while 31 SNPs were earmarked as damaging according to PolyPhen-2. A cluster of genes identified by the Condel tool as damaging was a total of 46 in the three T1D cases, while absent in the control. The type of substitution of the amino acids for each gene is listed in Table S1, supporting information. The presence of novel SNPs of < 2% was also noted within the exome data (Figure 3).

**Figure 4:**
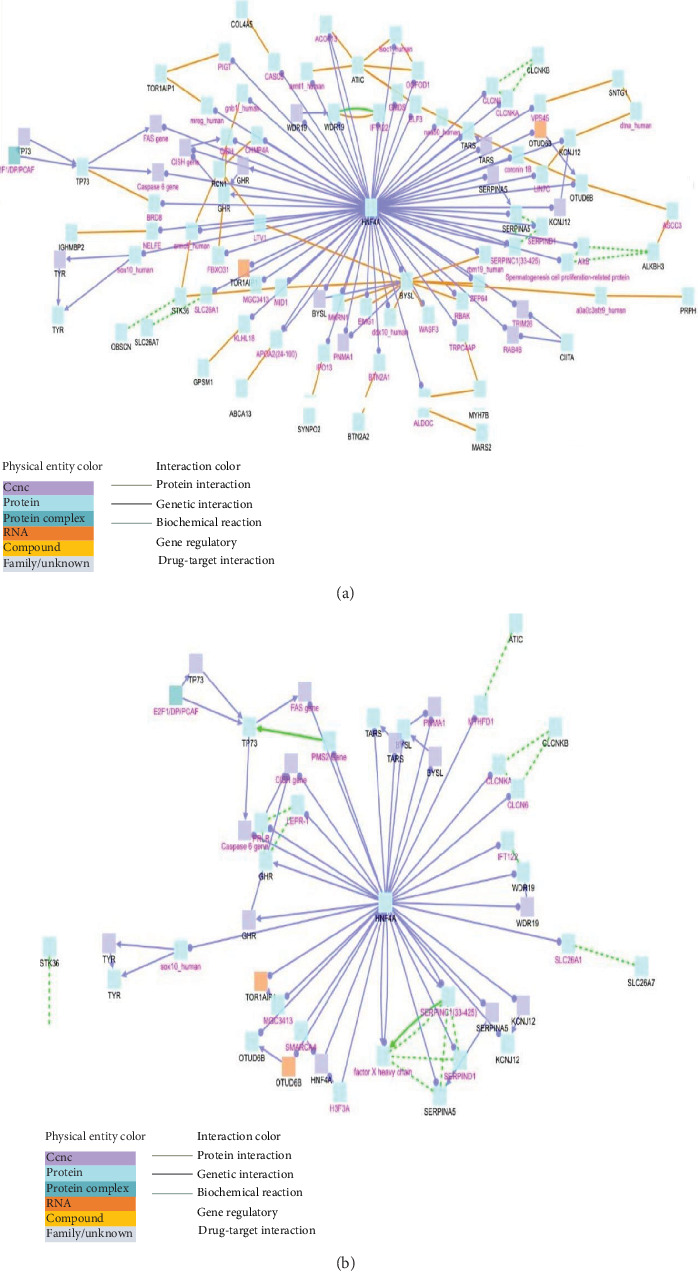
(a) Network of 46 genes extracted and classified as damaged by the Condel tool used as input to the ConsensusPathDB-human software. The figure places the HNF4A gene in the center as a hub demonstrating its wide range of molecular interactions and functionality across different classes of physical and biological molecules, including genes, proteins, DNA, RNA, and drug targets. (b) Network of the same 46 genes as input using the ConsensusPathDB-human software. Only gene–gene interaction was employed. The figure shows again the centrality position of HNF4A in the network.

**Figure 5:**
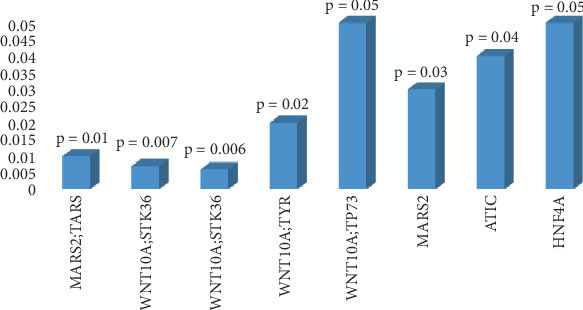
The top eight significant pathways with a *p* value of ≤ 0.05 based on damaged genes extracted from the exome data using KEGG, 2018. These show a clear association with T1D and tumorigenesis.

Several deleterious mutations in genes known to be related to T1D in other populations have been described, such as INS, HLA, CTLA4, PTPN22, and IFIH1. Several mutations in the heterozygous state of different types using different tools were found in all results in the INS gene. Most prominently, rs3842752 is classified as pathogenic by a SIFT score of 0.04. Nonsynonymous variants also in the heterozygous state were found in the CTLA4 and PTPN22 genes with no conclusive SIFT or PolyPhen-2 scores. However, IFIH1 had both high SIFT and PolyPhen-2 scores (Table 2). All changes were either A/G or C/T, a known signature of APOBEC/AID editing enzymes. Network analysis using ConsensusPathDB-human (http://cpdb.molgen.mpg.de/CPDB/cySVis-2016) predicted different types of interactions with HNF4A being centrally located in the network (Figure 4a,b). Of 40 pathways annotated in the KEGG database, a total of eight pathways were significantly overrepresented (*p* value ≤ 0.05) in target lists, including WNT, MARS2, TARS, STK36, TYR, TP73, ATIC, and HNF4A (Figure 5). The results of gene pathway showed a significant association (*p* ≤ 0.05) between several genes with known involvement in T1D or tumorigenesis (Table S2). Several genes that may be pathogenic or relevant to some complications of T1D were found to be below the significance threshold of *p* ≤ 0.05, possibly due to the limited sample size (Table 3).

Bioinformatic analysis showed several miRNAs known to regulate many mRNAs from pathways related to the immune system and T1D pathogenesis. The results showed that the three cases shared about seven of these miRNAs, miR-210-5p, miR-25-3p, miR-30a-3p, miR-503-5p, miR-27a-5p, miR-181a, and miR-320e, with strong associations with T1D and related functions (Table 4).

Two miRNAs (miR-105 and miR-518) identified by in silico analysis based on marginal or suggestive predictive values (*p* ≤ 0.057 and 0.038, respectively) were implicated subsequently by expression profiling, as potentially novel susceptibility factors for T1D (Table S3). Among genes targeted by miR-518 and miR-105, a significant association with the Transcription Factor 7–Like 2 (TCF7L2) genetic pathway was found. This transcription factor plays a key role in the Wnt signaling pathway. The protein has been implicated in blood glucose homeostasis. Others, like hydroxyacyl-CoA dehydrogenase, trifunctional multienzyme complex subunit alpha (HADHA), and GTPase (KRAS), significantly enriched in different types of cancer and targeted by miR-105 and miR-518, were also shown and are listed in Table S4. Based on the above findings of network centrality, HNF1A rs2464195 and HNF4A rs147638455 were genotyped in family siblings and 47 patients with T1D, in addition to 23 healthy controls (e.g., the parents and daughter), and the results are shown in Table S5. However, no significant difference was seen between cases and controls (*p* = 1 and *p* = 0.73). Allele and genotype frequencies of both variants are shown in Table S6; both variants are common in the T1D samples and controls, with a minor allele frequency (MAF) of 0.1363 and 0.14, while MAF in other populations, including Africans, ranged between 0 and 0.001101. From the point of view of genotype–phenotype association, this is not conventional in T1D, since data suggest that HNF4A genetic variants are not necessarily associated with maturity-onset diabetes of the young (MODY) either, because the onset of the disease for all cases was before the age of 9.

Expression levels were varied in HNF1A between cases and controls, and within cases, the fold change was 6.9, 1.5, and 1.8 for cases DA60, DA61, and DA62, respectively. Similarly marked changes were found in the expression profiles of the HNF4A; the diabetic cases express HNF4A 1.2-, 16-, and 20.67-fold higher than the control samples.

**Figure 6:**
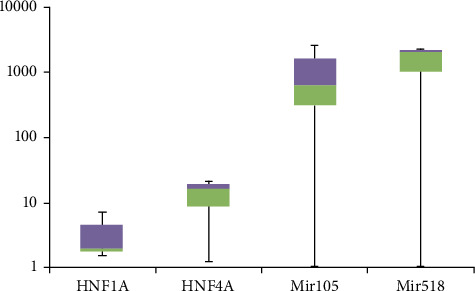
Box plot and whiskers diagram (logarithmic scale) showing the fold change in expression in HNF1A, HNF4A, and that of miR-105 and miR-518 using the Ct method. The experiments were performed in duplicate. The colors and lines indicate the five values: minimum, 25th percentile, median, 75th percentile, and maximum fold change for each set. The Livak equation was used to calculate the fold change for HNF1A, HNF4A, miR-105, and miR-518.

Expression of miR-105 and miR-518 was presented as box blots (Figure 6). In case DA60, the fold difference for miR-105 in expression was −512 lower than the control, whereas in cases DA61 and DA62, the fold changes were 2048- and 97-fold, respectively, higher than the controls.

In case DA60, the fold difference for miR-518 was −2048 lower than the control, while for cases DA61 and DA62, it was 6.9 and 256 times higher. It is obvious that DA60 expression profiles for both HNF1 and HNF4, as well as for the miRNA, are different from the two other siblings, consistent with differences in the clinical phenotype.

## 4. Discussion

Regardless of its type, diabetes mellitus in general is a multifactorial disease, with little known about its molecular epidemiology, molecular mechanisms, and loci that may be underlying its phenotype. The dilemma of assigning a phenotype to genotype is not only compounded by the sheer nature of a complex disease but more so by the unique milieu of African genetic diversity [16] and the large East African population size [17]. This family attests to that by the number of variants in each exome case of approximately 90,000 SNVs but also by layers of peculiarity within.

The exome data included several missense variants, which were defined as nonsynonymous substitutions, and about 175 nonsynonymous variants were shared between all cases. In many studies, half of the missense mutations found by genome sequencing, inferred as deleterious, correspond to nearly neutral mutations that have little impact on the clinical phenotype of individual cases [18].

Like many diseases of complex inheritance, the biology of T1D varies across the spectrum of genetic basis of susceptibility and inheritance. Some genes, like the INS gene, are reported to have mutations that lead to the production of structurally abnormal INS with reduced biological activity and receptor binding that causes diabetes mellitus [19]. Any defect in the INS gene or insulin growth hormone factor (IGF) may lead to the destruction of the INS-producing cells (*β*-cells), and it was thus the main target in various biological and genetic studies among T1D populations, as a candidate for a gene with major effect.

In a nontypical fashion, several other T1D candidates were recovered in one single family, perhaps a feature of the large African population size mentioned earlier, for example, the rs231775 missense mutation in CTLA4. Investigation of the role of CTLA4 in the regulation of T-cell immune responses has revealed that CTLA4 is a very important molecule involved in the maintenance of T-cell homeostasis [20]. It is thought that inherited changes in CTLA4 gene expression can increase T-cell self-reactivity and therefore play an important role in autoimmune diseases such as T1D (https://www.diapedia.org/type-1-diabetesmellitus/2104135127/ctla4). The nonsynonymous variant supports the importance of the gene among diabetic patients, as it is a negative regulator of T-cell activation and development and has been associated with susceptibility to several autoimmune diseases, including T1D [21].

Another example is the C1858T nonsynonymous variant (R620W, rs2476601) in the lymphoid Protein Tyrosine Phosphate Nonreceptor Type 22 (PTPN22) gene, encoding lymphoid-specific tyrosine phosphatase (LYP). This gene has been shown to be associated with several other autoimmune diseases, including rheumatoid arthritis, systemic lupus erythematosus, Graves' disease, Addison's disease, and myasthenia gravis, suggesting a general role of LYP in the autoimmune process [21], and the current exome analysis supports results obtained by other studies which showed a strong relationship between PTPN22 and T1D [22].

Results obtained from exome data revealed different types of mutations in the IFIH1 gene, some with SIFT and PolyPhen-2 scores of 0 and 1, respectively. IFIH1 is strongly associated with T1D and is expressed in human islets and *β*-cells. It is crucial for the immune response to enterovirus infection or exposure to synthetic dsRNA. Some studies suggest that one of the IFIH1 mechanisms that contributes to *β*-cell destruction in T1D is by increasing the local production of inflammatory cytokines and chemokines, thereby exacerbating islet immune cell infiltration [23].

HNF4A was one of the damaged genes that were extracted from the exome data. The variant was found among the siblings of a Sudanese family with T1D. HNF4A is a gene that acts as a switch that turns on and off other genes in the body. Changes in the HNF4A gene cause diabetes by reducing the amount of INS produced by the pancreas (http://www.diabetesgenes.org/content/hepatic-nuclear-factor-4-alpha-hnf4a). In general, HNF4A has been shown to be a positive transcriptional activator of many essential genes [24]; for example, HNF4a regulates genes involved in cholesterol, fatty acid, amino acid, and glucose metabolism and activates at least one other liver-enriched transcription factor [24].

Interestingly, several of the annotated variants were directly related to other types of diabetes mellitus, like gestational diabetes, MODY, and Type 2, but also to T1D. Henceforth, it may be possible to consider adopting future genetic classification in which all types fall under one umbrella (diabetes mellitus) but are different in terms of detection and treatment on a molecular basis. Accordingly, personalized molecular findings in patients may depend on how T1D and T2D share and overlap in select clinical and pathological features due to some common underlying genetic and molecular mechanisms [25]. What should also be considered is to rank the associated mutations according to their role in disease etiology. For instance, HNF1A is reported to play a minor role in the pathogenesis of Type 2 diabetes mellitus in Brazilian individuals [26], while HNF1A mutations are the most common cause of MODY in Europe, North America, and Asia [27]. According to genotyping results in this dataset and ClinVar criteria, the HNF1A G/A (rs2464195) variant is benign or likely benign.

Similarly, HNF4A encodes for hepatocyte nuclear factor and has an important role in pancreatic development and maintenance of beta-cell function, inflammation, and lipid metabolism [28]. These variants were found to be common in the Type 1 sample and the family analyzed but not in other populations like Europeans and even Africans. The delineation of common or rare is often critical in assigning a variant to a category of pathogenicity and is considered essential in personalized exome analysis. The allele frequency of the variant is inversely proportional to the effect size, normally expecting rare variants to have a larger effect size. That being said, the role of genetic changes within a system perspective should also be considered in such ranking, as should population frequencies, which could be quite variable even within the same country or ethnic group, as the comparison reveals in the current study.

Contrary to widely held concepts and approaches which emphasize the role of single genes, in the current family, the role of HNF4A seems pivotal in the network, implying functionality, despite its abundance in the control sample with a frequency of 0.17 and its relative commonness, in the Sudanese genomic databases. This functionality may be due to its activity—which has been shown to be regulated in several ways [29], including posttranscriptional modifications, such as phosphorylation [24] or acetylation [30], as well as protein–protein interactions with other factors [31]—and does not necessarily imply a mutation in the gene itself. It is a functionality that is corroborated by the slight increase in gene expression of both HNF1A and HNF4A in cases compared to controls, as changes in expression do not necessarily affect the same gene but affect other genes with different functions. What regulates the function of these genes remains to be verified. It is well established that miRNAs regulate INS production by directly or indirectly affecting the expression of key transcription factors, and they contribute to fine-tuning of hormone release by modulating the levels of important components of the beta-cell secretory machinery [32]. miRNAs may serve as points of crosstalk between signaling pathways, by integrating transcriptional inputs or by their functional regulatory output on different pathways [31]. MicroRNAs are involved in the pathogenesis of diabetes mellitus by affecting pancreatic *β*-cell functions, INS resistance, or both [33]. Many miRNAs have been implicated in normal pancreatic development and function. Moreover, given the complex interplay between many miRNAs in normal pancreatic cells, it is expected that aberrant miRNA expression or mutations could result in *β*-cell pathology. Several miRNAs are released into the bloodstream or expressed in blood cells and might be used as circulating biomarkers of T1D [15, 34]. This includes miR-25-3P [35, 36], miR-181a [35], and miR-27a-5p, which was found to be positively correlated with *β*-catenin, a key protein involved in Wnt signaling that is also one of the pathways in the current analysis [37]. miR-320e is a potential mediator of several angiogenic factors in diabetic myocardial microvascular endothelial cells (diabetes complications) [38].

Although we are not in the position to make a direct inference on such an effect between the susceptibility loci identified and miRNA expression, however, miR-518 was shown in some studies to be differentially expressed in placentas from patients with gestational diabetes mellitus (GDM) by activation of a receptor-*α* of the PPAR*α* gene [39]. The existence of TCF7L2, as one of the miR-105 target genes, within the analyzed data, supports the idea of diabetes as one disease regardless of the type. Variants of TCF7L2 were found to be associated with different types of diabetes, including T2D in Sudan [40].

Also, of interest here is the different expression profiles of patient DA60, where the fold change was shown to be downregulated as compared to the other siblings. Apparently, due to poor control of the disease, this patient suffers from short stature (Hashimoto's disease) and celiac disease, which are diseases of immunogenic origin like T1D. The chance of developing an autoimmune disease increases if the person has any other autoimmune diseases, as patients with T1D often suffer from celiac disease and Hashimoto's disease. Although the exact role of this differential expression is yet to be elucidated, the fact that the variation exists within siblings in the same family emphasizes the importance of adopting individualized approaches in the quest to understand disease pathogenesis.

Interesting are the types of mutations in key affected genes, all of which are TID candidates. The pattern (CT/AG) is suggestive of being an outcome of APOBEC/AID editing enzymes. These enzymes are known to have viral interactions that might act as triggers and may provide an alternative explanation for the role of viruses in T1D. Given the low statistical odds of encountering such mutational patterns in the above results of multiple T1D genes being potentially pathogenic and their position in a network-based analysis, all prompt an alternative approach to the etiology and pathology of T1D.

In conclusion, this single T1D family dataset and population cohort investigated, being limited though, enabled important insights into various types of interactions and genetic effects of single or combined nature, based on genomic analysis. Mutations in coding genes might not be enough to implicate a variant functionally, as shown in this family, and the analysis of whole genomes or large genomic sequences in an individualized manner is a necessity.

## Figures and Tables

**Table 1:** The clinical characteristics of the three Type 1 diabetes mellitus cases and the control in the investigated family. Celiac was positive in cases DA60 and DA62. TSH and T3 were above the normal range compared to case DA61 and the control, in addition to the increased level of microalbumin in case DA60. The patients had developed T1D before the age of 9 years. All patients were treated with insulin.

	**DA60**	**DA61**	**DA62**	**DA612 (control)**
Sex/age, year	M/23 years	M/25 years	F/12 years	F/27 years
Age at diagnosis, year	5 years	12 years	7 years	Healthy
Weight, kg^a^	35	52.5	27	59
Height, cm^a^	138	171	141	131
HbA1C, %^a^	11.4	10.6	11.6	6.5
C-Peptide, ng/mL^a^	< 0.01	< 0.01	< 0.01	3.60
GAD autoantibodies^a^	Negative	Negative	Negative	Negative
TSH, *μ*IU/mL^a^	1.8 (normal)	0.97 (normal)	7.1 (high)^b^	0.81 (normal)
FT3, pg./mL^a^	2.9 (normal)	2.85 (normal)	4.0 (high)	2.5 (normal)
FT4, ng/dL^a^	1.3 (normal)	1.01 (normal)	1.0 (normal)	0.9 (normal)
Celiac^a^	Positive^b^	Negative	Positive	Negative
Microalbumin, mg/L^a^	68 (high)	11	17	12
Treatment	Insulin	Insulin	Insulin	None
Complication	Celiac/stature	None	Celiac	None

^a^Upon the study.

^b^Detected upon diagnosis.

**Table 2:** Variant type, rsID, SIFT, and PolyPhen-2 values of the four major susceptibility genes associated with T1D found in members of the family. The common locus linked to T1D is the INS gene. Other immune-related genes (CTLA4, TPN22, and IFIH1) highlight the potential role of immunity in T1D.

**Gene**	**Variant**	**rsID**	**SIFT**	**PolyPhen-2**
INS	UTR3	rs3842752	0.04	—
CTLA4	Nonsynonymous	rs231775	0	0.16
PTPN22	Nonsynonymous	rs2476601	0	1
IFIH1	Nonsynonymous	rs3747517	0	1

**Table 3:** Distributions of major and minor alleles, significance, and type of mutation of the most prominent genetic variants associated with diabetes mellitus extracted from exome data.

**Gene**	**SNP**	**Chr**	**Major allele**	**Minor allele**	**Significance**	**Type of mutation**
HNF1A	rs2464195	12	G	A	Unknown	Missense
HNF4A	rs147638455	20	A	G	Uncertain	Missense
KLF11	rs34336420	2	C	T	Pathogenic	Missense
INS	rs3842752	11	G	A	Pathogenic	Missense
SLC30A8	rs13266634	8	C	T	Risk factor	Missense
OAS1	rs10774671	12	G	A	Risk factor	Splice
PTPN22	rs2476601	1	A	G	Risk factor	Missense
KCNJ11	rs5219	11	T	C	Risk factor	Missense
CTLA4	rs231775	2	A	G	Risk factor	Missense

Abbreviation: Chr, chromosome.

**Table 4:** The seven miRNAs strongly associated with Type 1 diabetes mellitus or its complications extracted directly from the exome data (PolymiRTS database v 3.0).

**MicroRNA**	**Function**	**Reference**
miR-210-5p	Strongly linked with the hypoxia pathway and is upregulated in response to hypoxia-inducible factors. It is also overexpressed in cells affected by cardiac disease and tumors	[12]
miR-25-3p	Negatively associated with residual beta-cell function	[13]
miR-30a-3p	Members of the miR-30 family have been found to be highly expressed in the heart. It has also been suggested that the TP53 protein may be a target of miR-30c and miR-30e. P53 expression levels were elevated upon knockdown of miR-30c and miR-30e	[13]
miR-503-5p	Genetic variants influencing circulating lipid levels and risk of coronary artery disease	[14]
miR-27a-5p	Implicated in cholesterol homeostasis and fatty acid metabolism. The miR-27 gene family has been shown to be downregulated during the differentiation of adipocytes	[13, 15]
miR-181a	Strongly significant with diabetes mellitus. Significant correlations between the expression of miR-181a and both adipose tissue morphology and key metabolic parameters, including visceral fat area, HbA1c, and fasting plasma glucose	[13]
miR-320e	The predicted genes targeted by miR-320 include IGF-1 and IGF-1R. The expression of IGF-1 and IGF-1R proteins decreased significantly in diabetic myocardial microvascular endothelial cells (MMVEC)	[12]

## Data Availability

All data supporting the study are included and referenced within the manuscript. Exome data from Illumina HiSeq 2000 are available at the Institute of Endemic Diseases server and are available from the corresponding author on reasonable request.

## Supporting Information

Supporting Information Additional supporting information can be found online in the Supporting Information section. Figure S1: Variants' statistics of exome data. This figure provides comprehensive variant statistics resulting from the exome sequencing data, including variant types, as well as sift, PolyPhen-2 functional score, damaged genes, and microRNAs. Table S1: List of 46 damaged genes found among cases only (extracted by the Condel tool). In most genes, the change did not affect their function. HGVS: Human Genome Variation Society nomenclature; 1KG: 1000 Genome. Table S2: The pathways of a group of damaged genes extracted from the exome data (KEGG, 2018). This table summarizes the biological pathways most affected by these gene variants, providing insights into a number of diseases associated with these genes. Table S3: Selection of microRNAs (Enrichr platform). MicroRNAs were selected based on significant differences in order to study expression levels. Table S4: Shows the target genes for miR-105 and miR-518 (DIANA tool, 2018). This table shows the target genes for miR-105 and miR-518 along with their associated biological pathways. Table S5: HNF4A (rs147638455) and HNF1A (rs2464195) genotypes among Type 1 diabetes mellitus cases and controls (e.g., the three cases and the three controls from the study family). No difference was seen between cases and controls for both loci (*p* = 0.73 and *p* = 1). Table S6: Allele frequency of HNF1A (rs2464195) and HNF4A (rs147638455) among the Sudanese population (patients and healthy controls), in addition to other populations, including Africans (ExAC Browser, 2016).
