# Supplementary material for: Exome Sequencing of a Type 1 Diabetes Mellitus Family Exposes Both Common and Individualized Rare Variants Contributing to Pathogenesis
Source: J Diabetes Res. 2025 Dec 2;2025:3346256. doi: 10.1155/jdr/3346256 (PMC12688644; doi:10.1155/jdr/3346256)
Supplement: Supporting Information — Additional supporting information can be found online in the Supporting Information section. Figure S1: Variants' statistics of exome data. This figure provides comprehensive variant statistics resulting from the exome sequencing data, including variant types, as well as sift, PolyPhen-2 functional score, damaged genes, and microRNAs. Table S1: List of 46 damaged genes found among cases only (extracted by the Condel tool). In most genes, the change did not affect their function. HGVS: Human Genome Variation Society nomenclature; 1KG: 1000 Genome. Table S2: The pathways of a group of damaged genes extracted from the exome data (KEGG, 2018). This table summarizes the biological pathways most affected by these gene variants, providing insights into a number of diseases associated with these genes. Table S3: Selection of microRNAs (Enrichr platform). MicroRNAs were selected based on significant differences in order to study expression levels. Table S4: Shows the target genes for miR-105 and miR-518 (DIANA tool, 2018). This table shows the target genes for miR-105 and miR-518 along with their associated biological pathways. Table S5: HNF4A (rs147638455) and HNF1A (rs2464195) genotypes among Type 1 diabetes mellitus cases and controls (e.g., the three cases and the three controls from the study family). No difference was seen between cases and controls for both loci (p = 0.73 and p = 1). Table S6: Allele frequency of HNF1A (rs2464195) and HNF4A (rs147638455) among the Sudanese population (patients and healthy controls), in addition to other populations, including Africans (ExAC Browser, 2016). [file 3346256.f1.pdf]

Supplementary data

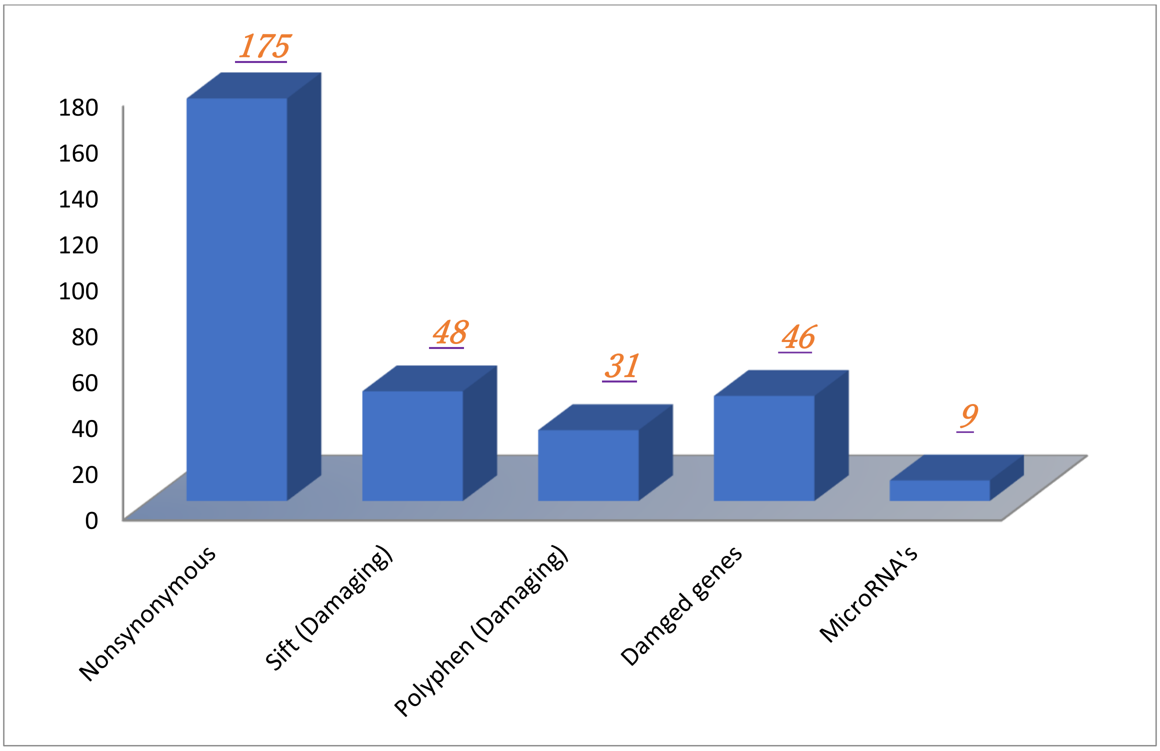

Figure S1. Variants' statistics of exome data.

Table S1. List of 46 damaged genes found among cases only (extracted by Condel tool). In most genes the change did not affect their function. HGVS: the Human Genome Variation Society nomenclature; 1KG: 1000 Genome

Gene	rsID	SIFT	PolyPhen-2	HGVS	1KG allele frequency (global)
ARHGAP10	rs112034189	0.34	0.274	NM_024605:c.G1861C:p.E621Q	-
ATIC	rs149185538	0.08	0.165	NM_004044:c.T1715C:p.I572T	0.0009
	-	0.19	0.957	NM_004044:c.A1751G:p.H584R	0.0005
MARS2	-	0.51	0.003	NM_138395:c.G1099A:p.V367M	-
MOGAT1	-	0.15		NM_058165:c.G352T:p.A118S	-
	rs1868024	0.41		NM_058165:c.T487C:p.S163P	0.23
BTN3A1	rs56161420	0.16	0.002	NM_001145008:c.G44A:p.R15H	0.13
	rs4712990	0.06	0.856	NM_001145008:c.C1210A:p.P404T	0.1
C10ORF58	rs72860701	0.27	0.307	NM_152290:c.C134T:p.T45I	0.02
MLPH	rs3751109	0.2	0	NM_001042467:c.T458C:p.L153P	0.17
	rs3751107	0.45	0	NM_001042467:c.G515A:p.G172D	0.17
	rs11883500	0.29	0.002	NM_001281473:c.C746T:p.T249I	0.13
	rs2292884	1	0	NM_024101:c.A1040G:p.H347R	0.33
	rs3817362	0.57	0.048	NM_001281473:c.T917C:p.V306A	0.36
	rs58256353	0.32	0.091	NM_001281474:c.C922T:p.P308S	0.06
SNTG1	-	0.01	0.999	NM_001287814:c.A784G:p.N262D	-
HELT	rs1078461	0.11	0.184	NM_001029887:c.C439G:p.L147V	0.12
MYH7B	rs11906160	0	0.200943	NM_020884:c.G73A:p.A25T	0.14
	rs2425015	1	0.437989	NM_020884:c.A3019G:p.K1007E	1
	-	0.05	0.455088	NM_020884:c.C3971A:p.T1324K	-
IGHMBP2	rs560096	0.72	0	NM_002180:c.T602C:p.L201S	0.7
	rs622082	0.31	0	NM_002180:c.A2011G:p.T671A	0.22
	rs2236654	0	0.512	NM_002180:c.C2080T:p.R694W	0.17
SERPINA5	rs6118	0.37	0.527	NM_000624:c.C164T:p.A55V	0.25
	rs6119	1	0	NM_000624:c.A313G:p.K105E	0.24
WDR19	rs75964850	0.04	-	NM_025132:c.G910A:p.V304I	0.03
GPSM1	rs60980157	0.09	0.002	NM_001145638:c.C1172T:p.S391L	0.19
ABCA13	rs17712293	0	-	NM_152701:c.T2300G:p.I767S	0.18
	rs17712299	0	-	NM_152701:c.T4618C:p.F1540L	0.11
	rs17661364	0	-	NM_152701:c.A6097G:p.N2033D	0.04
	rs1880736	1	-	NM_152701:c.C6533A:p.A2178E	0.78
	rs78334925	0	-	NM_152701:c.A6917C:p.K2306T	0.11
	rs2222648	1	-	NM_152701:c.C8020T:p.R2674W	0.83

	-	0.05	-	NM_152701:c.G11410C:p.E3804Q	-
	rs6583448	1	-	NM_152701:c.A13336G:p.K4446E	1
ALKBH3	rs2434470	0	0.997	NM_139178:c.C684G:p.D228E	0.16
GPBAR1	-	0	-	NM_001077191:c.C328T:p.R110C	-
CIITA	rs2229317	0.02	0.98	NM_000246:c.C133G:p.L45V	0.15
	rs8046121	0.58	0	NM_000246:c.A520G:p.R174G	0.99
	rs4774	1	0	NM_000246:c.G1499C:p.G500A	0.26
	rs7197779	0.3	0.398523	NM_001286403:c.A947G:p.Q316R	0.95
STK36	rs1863703	0.21	0.019	NM_001243313:c.A884G:p.K295R	0.15
	rs16859180	0	0.999	NM_001243313:c.C1429T:p.R477W	0.05
	rs1344642	0.16	0.892	NM_001243313:c.G1748A:p.R583Q	0.42
	rs6709303	1	0	NM_001243313:c.A1913C:p.Q638P	0.04
	rs55633575	0.27	0.001	NM_001243313:c.G2947A:p.V983I	0.04
	rs12993599	0.45	0.124	NM_001243313:c.G3272A:p.R1091Q	0.04
	rs73079079	0.59	0.966	NM_001243313:c.T3628G:p.C1210G	0.04
OBSCN	rs1771487	0.09		NM_001098623:c.A1505G:p.Q502R	0.7
	rs7532342	1		NM_001098623:c.T4523A:p.V1508D	1
	-	0.12		NM_001098623:c.C4796T:p.A1599V	-
	rs1188724	0.78		NM_001271223:c.A6143G:p.H2048R	0.72
	rs114195142	0		NM_001098623:c.C5278T:p.R1760C	0.01
	rs1188721	1		NM_001098623:c.T6318G:p.D2106E	0.72
	rs1188722	1		NM_001098623:c.T6346C:p.F2116L	0.69
	rs55797049	0.01		NM_001271223:c.G14228A:p.C4743Y	0.01
	rs1150912	0.76		NM_001098623:c.A13142G:p.H4381R	0.74
	rs1188732	0.17		NM_001098623:c.T13348C:p.C4450R	0.73
	rs116329268	0.01		NM_001098623:c.A13490T:p.Q4497L	0.02
	rs4653942	0.1		NM_001098623:c.G13601A:p.R4534H	0.34
	rs1188729	0.04		NM_001098623:c.C13925G:p.S4642C	0.73
	rs55940470			NM_001098623:c.C13925G:p.S4642C	0.01
	rs3795801	0.26		NM_001098623:c.G13996A:p.G4666S	0.18
	rs373610	0.38		NM_001098623:c.A14885G:p.D4962G	0.71
	rs500049	0.17		NM_001098623:c.C21515T:p.A7172V	0.56
RCN1	rs144307253	0.03	0.995	NM_002901:c.G274A:p.D92N	
HNF4A	rs147638455	0.03	0.386	NM_000457:c.A1387G:p.I463V	
TAS1R1	rs188357154	0.02	0.997	NM_138697:c.G1196T:p.G399V	0.0009
	rs10864628	0.78	0	NM_138697:c.A1039G:p.K347E	0.94
FRAS1	rs4859905	1		NM_001166133:c.A95G:p.D32G	0.52
	rs6848030	1		NM_001166133:c.A727G:p.I243V	0.01
	rs6838959	0		NM_001166133:c.C1286A:p.S429Y	0.01
	rs114373602	0.91		NM_025074:c.C6608T:p.T2203I	0.02
	rs76623027	0.34		NM_025074:c.G6691A:p.G2231R	0.02
	rs78404051	0.71		NM_025074:c.G6754A:p.A2252T	0.02
	rs7684722	1		NM_025074:c.A7132G:p.K2378E	0.98
	rs931606	1		NM_025074:c.G10696A:p.V3566I	0.51

OTUD6B	-	0		NM_016023:c.C755G:p.T252R	
TOR1AIP	rs2245425(splicing)	-			0.63
	rs609521	0.1	0.63	NM_001267578:c.C830G:p.P277R	0.59
	rs1281378	0	0	NM_001267578:c.T437C:p.M146T	0.61
PRPH	rs62636520	0.1	0	NM_006262:c.G829A:p.A277T	0.0.03
VPS13B	rs7833870	1	0	NM_017890:c.T7751C:p.V2584A	0.09
	rs140179844	0.23	0.021	NM_017890:c.G7783A:p.D2595N	0.0032
	rs6468694	0.61	0.985	NM_017890:c.G10294A:p.G3432R	0.13
OR8D4	rs17127947	0	0.903	NM_001005197:c.T164G:p.L55R	0.19
	rs17127950	0.01	0.912	NM_001005197:c.A274G:p.I92V	0.19
	rs7926767	0.21	0	NM_001005197:c.G398A:p.R133K	0.7
	rs7942047	1	0	NM_001005197:c.T848C:p.L283P	0.83
	rs7927385	1	0	NM_001005197:c.G893A:p.R298K	0.7
RBPJL	rs35032855	0.02	0.487	NM_001281448:c.G209A:p.R70Q	0.01
	-	0	0.991	NM_001281448:c.A568G:p.K190E	-
	rs35220957	0.34	0.707	NM_001281449:c.C1329A:p.S443R	0.01
	rs34078698	0.64	0	NM_001281449:c.T1439C:p.V480A	0.02
SYNPO2	rs17263971	0.05	0.514021	NM_001128933:c.A522C:p.Q174H	0.21
	rs7698598	1	0.279339	NM_001128933:c.A1717G:p.T573A	0.85
COL4A5	rs2272946	0.02	0.513806	NM_000495:c.T1331G:p.I444S	0.11
RP1	rs444772	0	0.001	NM_006269:c.G2615A:p.R872H	0.3
	rs35084330	0.02	0.996	NM_006269:c.G4784A:p.R1595Q	0.03
	rs414352	0.14	0	NM_006269:c.T5071C:p.S1691P	0.3
TYR	rs1042602	0.07	0.919	NM_000372:c.C575A:p.S192Y	0.18
PCCA	rs61749895	0.01	0	NM_001127692:c.G1573T:p.V525F	0.0032
WNT10A	-	0.19	0.97	NM_025216:c.A557C:p.K186T	
BTN2A2	rs73736234	0	0.533259	NM_001197237:c.C11T:p.A4V	0.01
	rs57038103	0	0.999	NM_001197237:c.C115G:p.P39A	0.01
	rs111615265	0	0.999	NM_001197239:c.A749G:p.Y250C	0.0009
	rs73736249	0.36	0	NM_001197239:c.A908C:p.K303T	0.01
SLC26A7	rs139871019	0.04	0.001	NM_052832:c.G400A:p.V134M	0.0027
CMYA5	rs16877109	1		NM_153610:c.A191G:p.Y64C	0.29
	rs1366271	1		NM_153610:c.G1046A:p.G349D	0.24
	rs16877124	1		NM_153610:c.G1772A:p.G591D	0.28
	rs57544556	0.73		NM_153610:c.A1951C:p.S651R	0.28
	rs6893869	0.51		NM_153610:c.T3017C:p.V1006A	0.29
	rs62621915	0.52		NM_153610:c.T3112C:p.F1038L	0.16
	rs4704585	1		NM_153610:c.C3884T:p.A1295V	0.56
	rs1428223	1		NM_153610:c.C4700A:p.A1567E	0.29
	rs1428224	0.84		NM_153610:c.T4795G:p.S1599A	0.29
	rs1428225	1		NM_153610:c.A5161G:p.I1721V	0.29
	rs16877151	1		NM_153610:c.A5758G:p.S1920G	0.29
	rs6859595	1		NM_153610:c.G6784C:p.V2262L	0.28
	rs192180434	0.03		NM_153610:c.G7479T:p.K2493N	0.0023
	rs2278239	0.17		NM_153610:c.A8718C:p.K2906N	0.28
	rs142926727	0.05		NM_153610:c.C10556A:p.P3519Q	0.0027
TP73	rs61737710	0.02	0.154	NM_001126240:c.G826A:p.A276T	0.01

CACNA1H	rs61734410	0.12		NM_001005407:c.C1919T:p.P640L	0.45
	rs1054645	0.16		NM_001005407:c.G6212A:p.R2071H	0.73
KCNJ12	rs1657738	0.06	0.157	NM_001194958:c.C44T:p.S15L	0.5
	rs74880280	0.08	0.002	NM_001194958:c.G106T:p.V36L	0.14
	rs78117732	0.03	0.181	NM_001194958:c.G128A:p.R43H	0.5
	rs1714865	0.54	0.003	NM_001194958:c.A167C:p.E56A	-
	rs73979893	1	0.001	NM_001194958:c.G213A:p.M71I	0.5
	rs76265595	0.01	1	NM_001194958:c.G415A:p.E139K	-
	rs76518282	0.02	0.408	NM_001194958:c.C425A:p.T142N	-
	rs75029097	0.01	0.998	NM_001194958:c.G433A:p.G145S	-
	rs1714864	0.01	1	NM_001194958:c.C467T:p.P156L	0.5
	rs73313922	0.42	0.995	NM_001194958:c.G517A:p.D173N	-
	rs73979896	0.04	0.003	NM_001194958:c.C554T:p.A185V	-
	rs72846667	0.09	0.086	NM_001194958:c.C631T:p.L211F	-
	rs77048459	1	0.008	NM_001194958:c.G715A:p.E239K	-
	rs4985866	0.65	0	NM_001194958:c.A745G:p.I249V	-
	rs74801394	0.18	0.99	NM_001194958:c.G906T:p.M302I	-
	rs80203231	0.04	0.041	NM_001194958:c.C1028T:p.S343L	-
	rs1612176	0.12	0.001	NM_001194958:c.C1113G:p.S371R	-
	rs73979902	0.09	0.101	NM_001194958:c.G1214T:p.S405I	-
	rs5021699	0.06	0.992	NM_001194958:c.A1289G:p.E430G	0.37
TARS	rs61734318	0.02	0.004	NM_152295:c.C2115G:p.I705M	0.0027
	rs112666038	0.01	0.79	NM_152295:c.G1738T:p.G580C	0.03
BYSL	rs2296916	0.03	0.256	NM_004053:c.G307A:p.E103K	0.08
	rs41273804	0.04	0.065	NM_004053:c.G736A:p.A246T	0.0005
	rs3828855	0.93	0	NM_004053:c.C1276T:p.P426S	0.01
CLCNKB	rs2015352	0.01	0.693	NM_000085:c.G80T:p.R27L	0.6
	rs5256	0.1	0.008	NM_000085:c.A262C:p.S88R	0.03
	rs12140311	0.1	0.003	NM_001165945:c.A934T:p.T312S	0.09
	rs5253	0.78	0.002	NM_001165945:c.T1178C:p.M393T	0.83
	rs2275166	1	0	NM_001165945:c.A1225G:p.K409E	0.7
	rs5255	0.02	0.964	NM_001165945:c.C1469T:p.S490L	0.01
GHR	rs6182	0.02	0.63	NM_001242460:c.G1253T:p.C418F	0.04
	rs6180	0.01	0.85	NM_001242460:c.A1564C:p.I522L	0.44
	rs6184	0.1	0.001	NM_001242460:c.C1669A:p.P557T	0.04

Table S2. The pathways of a group of damaged genes extracted from the Exome data (KEGG, 2018)

**Term**	**Gene**	***p-value***
Aminoacyl-tRNA biosynthesis_Homo sapiens_hsa00970	MARS2;TARS	0.01054
Basal cell carcinoma_Homo sapiens_hsa05217	WNT10A;STK36	0.007415
Hedgehog signaling pathway_ Homo sapiens_hsa04340	WNT10A;STK36	0.006163
Melanogenesis_Homo sapiens_hsa04916	WNT10A;TYR	0.02312
Hippo signaling pathway_ Homo sapiens_hsa04390	WNT10A;TP73	0.05024
Selenocompound metabolism_ Homo sapiens_hsa00450	MARS2	0.03922
One carbon pool by folate_ Homo sapiens_hsa00670	ATIC	0.04599
Maturity onset diabetes of the young_ Homo sapiens_hsa04950	HNF4A	0.05937

Table S3. Selection of MicroRNAs (Enrichr platform)

Index	Name	P-value
1	AGCGCTT,MIR-518F,MIR518E,MIR-518A ⟵	0.03888
2	GTCAACC,MIR-380-5P	0.04732
3	GTGTCAA,MIR-514	0.1259
4	GCATTTG,MIR-105 ⟵	0.05726
5	AGGAGTG,MIR-483	0.1355
6	AACTGAC,MIR-223	0.1946
7	TGAGATT,MIR-216	0.2104
8	ATGCTGG,MIR-338	0.2243
9	AACATTC,MIR-409-3P	0.2726
10	TTGGAGA,MIR-515-5P,MIR519E	0.2806

Table S4. Shows the target genes for MiR-105 & MiR-518(Diana tool,2018)

**KEGG pathway**	***p-value***	**target genes**	**Name of the miR.**
Fatty acid elongation (hsa00062)	2.84762420047e-17	HADHA	MiR-518
Fatty acid metabolism(hsa01212)	3.94816322505e-13	HADHA	MiR-518
Fatty acid degradation(hsa00071)	3.67526295534e-12	HADHA	MiR-518
Cell adhesion molecules(CAMs)(hsa04514)	0.0275469959952	F11R	MiR-518
Glycosphingolipid biosynthesis-lacto and neolacto series(hsa00601)	2.06917475723e-05	FUT4	MiR-105
Proteoglycans in cancer(hsa05205)	0.000194168682757	MDM2,GRB2,MAPK1,FGF2 ,KRAS GRB2, MAPK1, FGF2	MiR-105
Prostate cancer(hsa05215)	0.00206717750696	TCF7L2, CDKN1B, KRAS, CREB3L2, MAPK1, MDM2	MiR-105
Estrogen signaling pathway(hsa04915)	0.00561472293934	KRAS, CREB3L2, SP1, MAPK1, GRB2.	MiR-105
Thyroid cancer (hsa05216)	0.00561472293934	TCF7L2, KRAS, MAPK1	MiR-105
MicroRNAs in cancer (hsa05206)	0.00679442431212	KRAS, MAPK1, GRB2, MDM4, GLS, CDKN1B, MCL1, MDM2	MiR-105
Chronic myeloid leukemia (hsa05220)	0.019024192718	KRAS, MAPK1, GRB2, MDM2, CDKN1B	MiR-105
Endometrial cancer (hsa05213)	0.0192018777487	TCF7L2, KRAS, MAPK1, GRB2	MiR-105
Signaling pathways regulating pluripotency of stem cells.(hsa04550)	0.0254141429755	ZFHX3, KRAS, MAPK1, GRB2, FGF2, ACVR2B	MiR-105
Acute myeloid leukemia (hsa05221)	0.0261197507318	KRAS, MAPK1, GRB2, TCF7L2	MiR-105
Glioma(hsa05214)	0.0275336638215	KRAS, MAPK1, GRB2, MDM2	MiR-105
D-Glutamate and D-glutamate metabolism (hsa00471)	0.0332047935151	GLS	MiR-105
Choline metabolism in cancer (hsa05231)	0.0462774600203	KRAS, MAPK1, GRB2, SP1	MiR-105

Table S5. HNF4A (rs147638455) and HNF1A (rs2464195) genotypes among type 1 diabetes mellitus cases and controls (including the three cases and the three controls from the study family). No difference was seen between cases and controls for both loci (P = 0.73 and P = 1)

	**HNF4A**			**HNF1A**		
	**A/A**	**A/G**	**G/G**	**G/G**	**G/A**	**A/A**
**T1D (N = 50)**	43	7	0	44	6	0
**Controls (N = 23)**	19	4	0	20	3	0
**P- value**	0.73			1		

Table S6. Allele frequency of HNF1A (rs2464195) and HNF4A (rs147638455) among Sudanese population (Patients and healthy controls) in addition to other populations including Africans ((ExAC Browser, 2016)

Population	Allele frequency of HNF1A variant (rs2464195)	Allele frequency of HNF4A variant (rs147638455)
East Asian	-	0
African	-	0
European (Finnish)	-	0
South Africa	-	0.0001211
European (non-Finnish)	-	0.0004356
Latino	-	0.0006911
Other	0.3596	0.001101
Sudanese T1D individuals	0.1363	0.14
Sudanese healthy	0.869	0.17
Genome bank data (Sudanese)	0.475	0.1125
